# Large Scale Library Generation for High Throughput Sequencing

**DOI:** 10.1371/journal.pone.0019119

**Published:** 2011-04-27

**Authors:** Erik Borgström, Sverker Lundin, Joakim Lundeberg

**Affiliations:** Science for Life Laboratory, Royal Institute of Technology (KTH), School of Biotechnology, Division of Gene Technology, Solna, Sweden; Yale School of Medicine, United States of America

## Abstract

**Background:**

Large efforts have recently been made to automate the sample preparation protocols for massively parallel sequencing in order to match the increasing instrument throughput. Still, the size selection through agarose gel electrophoresis separation is a labor-intensive bottleneck of these protocols.

**Methodology/Principal Findings:**

In this study a method for automatic library preparation and size selection on a liquid handling robot is presented. The method utilizes selective precipitation of certain sizes of DNA molecules on to paramagnetic beads for cleanup and selection after standard enzymatic reactions.

**Conclusions/Significance:**

The method is used to generate libraries for de novo and re-sequencing on the Illumina HiSeq 2000 instrument with a throughput of 12 samples per instrument in approximately 4 hours. The resulting output data show quality scores and pass filter rates comparable to manually prepared samples. The sample size distribution can be adjusted for each application, and are suitable for all high throughput DNA processing protocols seeking to control size intervals.

## Introduction

The new sequencing technologies are reshaping the field of research in genome biology [1], [2], [3], [4], [5]. With the latest next generation sequencing platforms, such as the Illumina HiSeq 2000 and Life Technologies SOLiD4 capable of generating over 100 giga bases of data per run, the need for fast sample processing are continuously increasing. Further, the intricacy of instrument handling and sample processing has led to the development of large sequencing centers [6], capable of running large projects using several instruments simultaneously, making scalable library generation processes essential. In addition, smaller target sequence populations, such as transcriptome sequencing and exome sequencing, are even more dependent on sample multiplexing due to the high number of sample preparations needed to balance the throughput of the systems. Automating the sample processing for massive sequencing not only addresses these needs, but also stands to improve robustness and decrease the risk of human error [7], [8], [9], [10].

The preparation of DNA for next generation sequencing usually consist of four main operations, namely; (1) fragmentation, usually performed by mechanical shearing of the DNA such as high pressure or ultrasound treatment, (2) repair, modification and ligation of adapters, are all enzymatic steps preparing the sheared DNA by addition of universal sequences at the fragment ends thereby enabling amplification and hybridization of the sequencing primers, (3) size selection of DNA molecules with a certain length optimal for the current application or instrument and lastly (4) enrichment for DNA molecules with successfully ligated adapters [11].

Protocols of automated library preparation to the new generation of sequencers have been described recently [9], [10], [12]. Previous methods cover enzymatic reactions and the clean-up afterwards, although a flexible and automated alternative to the time-consuming agarose gel electrophoresis separation used for narrow size selection of libraries is still missing. Stand-alone commercial systems have very recently emerged targeting the problems of manual gel separation, LabChip XT (Caliper) and Pippin Prep (Sage Science). However, these systems require extra instrumentation not easily integrated in a fully automated workflow.

**Figure 1:**
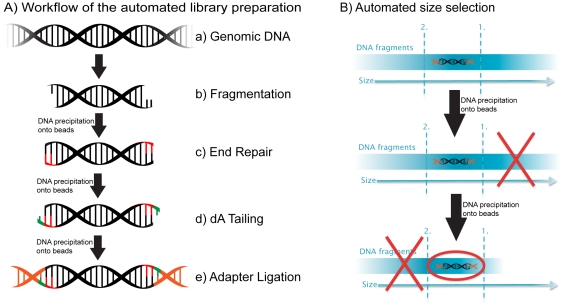
Flowchart of the sample preparation. A) Steps a through e explain the main steps in Illumina sample preparation, a) the initial genomic DNA, b) fragmentation of genomic DNA, c) end repair, d) addition of A bases to the fragment ends and e) ligation of the adaptors to the fragments. B) Overview of the automated the size selection protocol presented here. The first precipitation discards fragments larger than the desired interval. The second precipitation selects all fragments larger than the lower boundary of the desired interval.

In this study an automated protocol for preparation of samples prior to massively parallel sequencing is described to prepare DNA for paired end sequencing on the Illumina HiSeq 2000 instrument. The workflow (Figure 1) is demonstrated by generating libraries for de novo as well as re-sequencing projects, and validated by comparison to the standard manual procedures. The method utilizes precipitation of DNA on to carboxylic acid coated paramagnetic beads as a substitute for the spin columns used in the manual standard protocol, and a double sequential bead precipitation procedure replaces the manual agarose gel excision (Figure 1B). All precipitations utilize addition of poly-ethylene-glycol (PEG) and NaCl to the DNA sample. Details about this procedure and automation thereof can be found in the earlier publication on automatic library preparation for the GS FLX Titanium sequencing system [9].

## Methods

### Adaption of the standard protocol to automatic platform

The initial fragmentation of genomic DNA was performed by Adaptive Focused Acoustics on a Covaris S2 instrument (Covaris) instead of the standard nebulization method described in the manufacturer's protocol [6], [13], [14], [15]. The Covaris fragmentation method has been shown both to produce narrow fragment distributions as well as giving a better recovery of sample [6]. The automated version of the remainder of the protocol consists of two separate parts, both performed on a Magnatrix 1200 Biomagnetic Workstation (Nordiag). The first part of the protocol performs the enzymatic end repair, dA tailing and adapter ligation steps of the standard protocol (Paired-End Sample Preparation Guide, Illumina). While the second part performs size selection of ligation products replacing the gel cut procedure in the standard protocol. The two-part protocol design was chosen to promote flexibility in the protocol, enabling other methods for, or skipping of, the size selection step. Concentrations, volumes and incubation times used for the enzymatic reactions were set according the standard protocol. The intermediate spin column steps were replaced by PEG precipitation of DNA on to My One carboxylic acid coated paramagnetic beads (Invitrogen), using 15% PEG 6000 (Merck), 0,9 M NaCl and 10 minutes incubation time [9]. This setup enables selective precipitation of DNA molecules larger than 100 base pairs (bp) while smaller molecules such as nucleotides, non-ligated adaptors etc can be washed away.

The size selection part of the automated protocol utilizes two PEG solutions to perform sequential precipitations of DNA molecules. The concentration of the first PEG solution is chosen so that it, when added to the sample, enables precipitation of all molecules longer than the desired upper limit of the interval to be selected. The beads with the undesired molecules are discarded and the second PEG solution is added to the precipitation reaction solution, still containing all DNA molecules shorter than the upper length cut-off. The second PEG solution is chosen so to that it, when mixed with the supernatant from the first precipitation reaction, increases the PEG concentration of the supernatant enabling precipitation of all molecules longer than the lower limit of the interval to be selected. The beads are washed and DNA molecules within the desired size interval are eluted. Different shape and size of selected intervals can be obtained by varying the PEG concentrations for the two precipitation reactions. Following size selection the eluted DNA molecules were PCR enriched as described in the standard protocol and the size distribution and concentration of the final libraries were evaluated on the Agilent Bioanalyzer electrophoresis station and Qubit Quant-iTds DNA High Sensitivity (Invitrogen).

The range of the automated size selection protocol was assessed by precipitation of six different size intervals from the same pool of fragmented lambda genomic DNA. The PEG concentrations used can be found in Table S1. Two of the double precipitation reactions were performed in 5 duplicates to assess the robustness of the method. All products were evaluated on the Agilent Bioanalyzer using the High Sensitivity kit.

### Library preparations

All prepared samples started with 3 µg of DNA and were fragmented identically using the Covaris system. Following fragmentation the manual library preparations were performed as specified in the standard protocol, excluding the second agarose gel separation. Three libraries of spruce genomic DNA were prepared manually with insert sizes of about 190 bp, 320 bp and 700 bp respectively, for comparison with the automatically generated libraries.

The automatic library preparation protocol was used to prepare two spruce samples as well as three human cancer cell line samples (A-431 [16] and U-2 OS[17]). Two of the samples, one of each kind, were prepared with NEBNext DNA Sample Prep Master Mix Set 1 (New England Biolabs) reagents instead of the paired end sample preparation kit (Illumina) specified in the standard protocol. To be able to assess the effect of the automatic size selection, one of the automatically prepared cancer cell line samples was manually size selected by agarose gel separation as specified in the standard protocol. Fragmented samples and generated libraries were all evaluated using either the High Sensitivity or DNA 7500 kit for the Bioanalyzer.

Cluster generation of the prepared samples was performed using a HiSeq Paired-End Cluster Generation kit according to manufacturers instructions. Flow cells were clustered with one library and 1% phiX control library spike inper lane. The 320 bp manual library was prepared with final concentrations of 6, 7 and 8 pM and loaded in lane 1–3. The concentration of all automatically generated libraries loaded in lane 4–8 was 7 pM. The 190 bp, 320 bp and 700 bp manual libraries were also used in later instrument runs, with concentrations varying between 6–11 pM.

Sequencing of the clustered flow cell was preformed according to manufacturer’s instructions with settings for generation of 2×76 paired end reads.

### Data analysis

**Figure 2:**
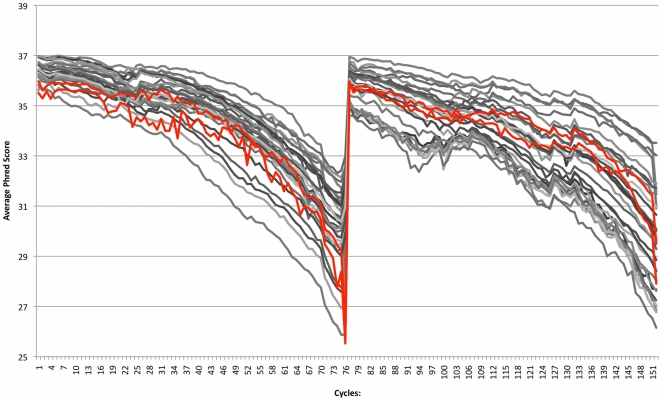
Average base call quality per cycle. Quality scores per cycle of 30 HiSeq 2000 lanes sequenced with manually (grey) and automatically (red) prepared spruce samples.

For all lanes the run statistics data such as percentage of passed filter clusters, Phred scores, cluster density and phiX error rates were obtained from the HiSeq Control Software. Further, additional data from 25 sequenced lanes of spruce, with varying insert size and cluster density (Table S2), were extracted from the instrument sequence files and used to compare the automated library generation method to manual preparations (Figure 2 and Figure S1). The reads from lanes 5, 6 and 8 corresponding to human cancer cell line samples were mapped to the human reference genome (hg19) by ELAND (Illumina). A 1% subset of the successfully mapped pairs were extracted and used to generate insert size distribution plots.

## Results and Discussion

The manual preparation of samples for sequencing is a very work demanding process. Four samples can take several days for a well-trained technician to prepare [7]. Although larger number of samples is possible to prepare, this will increase the risk of contamination and loss of quality in the final library. The protocol described in this study resolves the trade off between quality and quantity resulting in an increase of throughput to 12 samples in 6 hours (including 1 hour of hands on time). Compared to manual handling of 12 samples the hands on time of the automated protocol is less than the time needed for the agarose gel separation procedure alone. The enzymatic part of the automated protocol has an execution time of 3 hours and 45 minutes and the size selection part takes 30 minutes. Fragmentation of input sample, enrichment and evaluation of final libraries are considered to have equal throughput for manual and automatic procedures and are therefore not considered in the comparison. Illumina recently released their new TruSeq protocols for more high throughput library preparation. Although these protocols enable simultaneous preparation of up to 96 samples, the size selection is still done by manual agarose gel separation. The new protocols make use of reagent mixes and containers better suited for automation and adaptation of the here presented method to the new protocols could therefore further increase throughput and lower the required hands on time.

**Figure 3:**
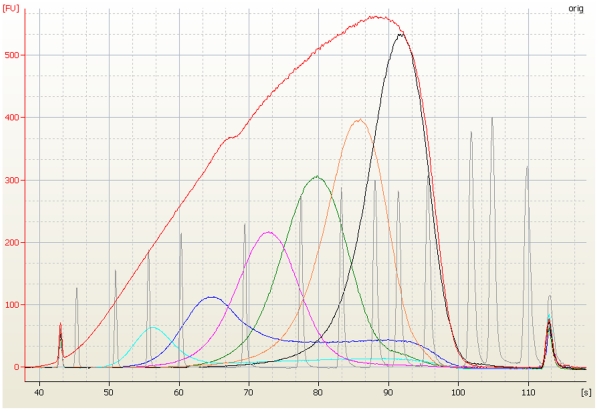
Automated size selection method. Six different size intervals were selected from the same fragmented sample pool (red) resulting in discrete population sizes ranging between 200–700 bp in average length and about 100 bp wide.

The automated size selection protocol has been used to select six different size intervals during one run (Figure 3). The different intervals were achieved by varying the PEG concentrations in the precipitation reactions. Some of the lower size intervals, average sizes of 200 and 300 bp in Figure 3, show a “tail” of larger fragments that have not been sufficiently removed. This has been observed to be an effect of the starting size distribution, and could be resolved by fragmenting differently. The five duplicates performed of the 500 bp and 600 bp size selections showed good reproducibility (Figure S2).

### Evaluation of libraries

**Figure 4:**
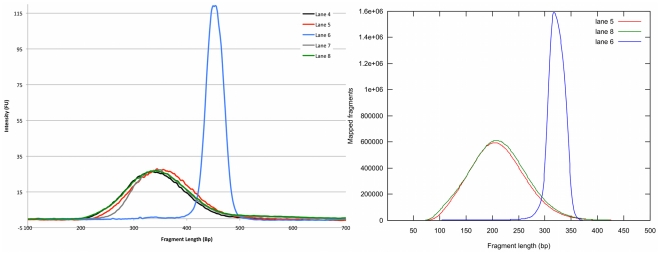
Size distribution of libraries. **a**. Bioanalyzer traces of generated libraries. Lane 4, 5, 7 and 8 correspond to libraries generated using the automatic size selection protocol. Lane 6 (blue) has been prepared using ordinary agarose gel selection. **b**. Insert size distributions of human cancer cell line libraries (lane 5, 6 and 8) acquired after mapping the reads to the human genome.

Automatic libraries showed good robustness in terms of size distribution and yield prior to sequencing. The libraries yielded final concentrations with a mean of 23.5 ng/µl and a standard deviation of 0.7 as determined by Qubit measurements. Bioanalyzer traces of the libraries show well-defined and reproducible traces of the libraries (Figure 4a).

### Evaluation of sequencing data

The cluster densities of the automatically generated libraries were generally higher than the manual ones. This effect can be explained either by a larger proportion of amplifiable molecules in the automated samples or a smaller average insert size of the automatic libraries.

From the average base call quality per cycle, based on 30 sequenced lanes from the same input DNA, we conclude that the variation between automatically and manually prepared samples are within the normal variation of the system (Figure 2, showing the two automatic spruce lanes and all lanes loaded with manual spruce libraries, 190–700 bp insert size). This is also the case when comparing lane passed filter (PF) rates and the percentage of PF reads where the average base call quality is above Q30, for lanes with similar number of generated reads (Figure S1). We find that the quality of base calls is proportional to the increase of cluster density, at a rate dependent on the sequencing run and average insert size of the libraries loaded. When the cluster density is almost twice the manufacturers recommendation we find that a large number of PF reads with satisfying quality are generated (lane 8, Table 1). The automatic library that was size selected by the standard procedure show lower cluster density and therefore also a higher PF rate and percentage of basecalls above 30 (>Q30%). Still the PF rates as well as the >Q30%, varies as much between the lanes run with the manual libraries as between manually and automatically prepared libraries. The libraries prepared with reagents from NEB (lane 7 and 8) show very similar performance to the corresponding sample prepared with standard reagents (Table 1).

**Table 1:** Sequencing run information.

Lane	Sample	Note	Conc. (pM)	Clusterdensity	# seq. pairs	# seq. pairs PF	>Q30 (%)	PF (%)
1	Spruce	Manual	6	400	73 768 362	66 246 185	89	90
2	Spruce	Manual	7	445	81 981 446	73 154 482	88	89
3	Spruce	Manual	8	488	89 947 283	79 179 058	87	88
4	Spruce	Auto	7	633	116 675 822	98 821 657	84	85
5	U-2 OS	Auto	7	644	118 762 987	99 614 234	85	84
6	U-2 OS	Auto, Manual gel cut	7	504	92 847 597	80 785 904	87	87
7	Spruce	Auto, NEB	7	653	120 322 872	101 014 779	84	84
8	A-431	Auto, NEB	7	718	132 257 940	106 038 199	84	80

Input parameters and result statistics from clustering and sequencing performed on manually and automatically prepared DNA libraries from plant and human sources.

The 1% phiX spike in all lanes functions as a positive control and should not affect the libraries it is loaded with. The phiX error rate cannot be used as a direct measure of library quality, it does however give information of the rate of accuracy of the sequencing reaction which can be affected by the library loaded. All phiX error rates are below the manufacturers threshold and lanes with similar cluster density show similar error rates seemingly independent of the type of the loaded library (Data not shown).

When mapped to the human genome the samples prepared with automatic size selection give an insert size distribution approximately two times wider than the ones prepared by the manual procedure (Figure 4b) showing good concordance to Bioanalyzer traces of the libraries prior to sequencing (Figure 4a).

### Size distribution of sequencing libraries

The automated size selection method described produces flexible and controllable size intervals, but the distribution obtained is approximately twice as wide as the manual gel separation. There is a trade off between yield and distribution width possible to obtain using this method. In theory, it should be possible to further control the distribution with the described method. The traces shown were the most suitable approach for the workflow needed, combining high yield (approximately 60%, Figure S2) and a distribution suitable for generating productive and good quality clusters to sequence. For certain applications, defining the distribution further could be necessary to alleviate downstream data processing, e.g. for applications such as structural variation detection where insertions or deletions lie close to the insert size mean, or where high resolution of the breakpoints are important. Currently, these applications could benefit from a manual gel separation. Commercial systems also targeting the problems of manual gel separation have recently emerged LabChip XT (Caliper) and Pippin Prep (Sage Science). Although these systems show a tighter insert size distribution than the method presented here, they are currently limited to 4 samples at a time. In cases where tighter insert size distribution outweigh the importance of sample throughput, these systems will contribute significantly.

In summary we have described an automated high throughput protocol for the preparation of samples for massively parallel sequencing. The libraries were sequenced using the HiSeq2000 system and comprehensively compared to manual procedures. A scalable automated non-gel based method for size selection of DNA molecules have been designed to replace the laborious and time consuming agarose gel separation step that dramatically increases sample throughput for massive sequencing, and are suitable for all similar DNA processing protocols demanding high throughput and a controllable size interval. The protocols described have also been used by other in-house projects to generate both indexed and exome capture libraries by exchanging the oligonucleotides used during adapter ligation and PCR. A modified version of the protocol is currently being tested for the SOLiD (Life Technologies) library preparation. The throughput of the described protocol is currently only limited by the instrument used and a larger liquid handling robot equipped with a 96-tip head could increase the throughput per run to 96 samples or more. This strategy constitutes a general approach to balance the increasing data throughput of the instruments for the preparation of samples for large scale sequencing projects.

## Supporting Information

Figure S1 **Effect of different clustering parameters and instrument runs.** Passed filter rates and percentage of PF read base calls that have quality scores above 30 for HiSeq 2000 lanes with manually and automatically (red edge) prepared spruce samples. The colors of the markers denote different instrument runs. Insert size and concentration used for the cluster generation can be found in the label for each pair of data points.
(TIF)

Figure S2 **Robustness of the automatic size selection method.** Two intervals (500 bp and 600 bp) were size selected and repeated five times.
(TIF)

Table S1 **PEG concentration of the two solutions used for each size interval in** **Figure 1.**
(DOCX)

Table S2 **Parameters and results for the 25 extra lanes included in** **Figure 2** **and Figure S2.**
(DOCX)
