# Supplementary figures and images for: Large Scale Library Generation for High Throughput Sequencing
Source: PLoS One. 2011 Apr 27;6(4):e19119. doi: 10.1371/journal.pone.0019119 (PMC3083417; doi:10.1371/journal.pone.0019119)

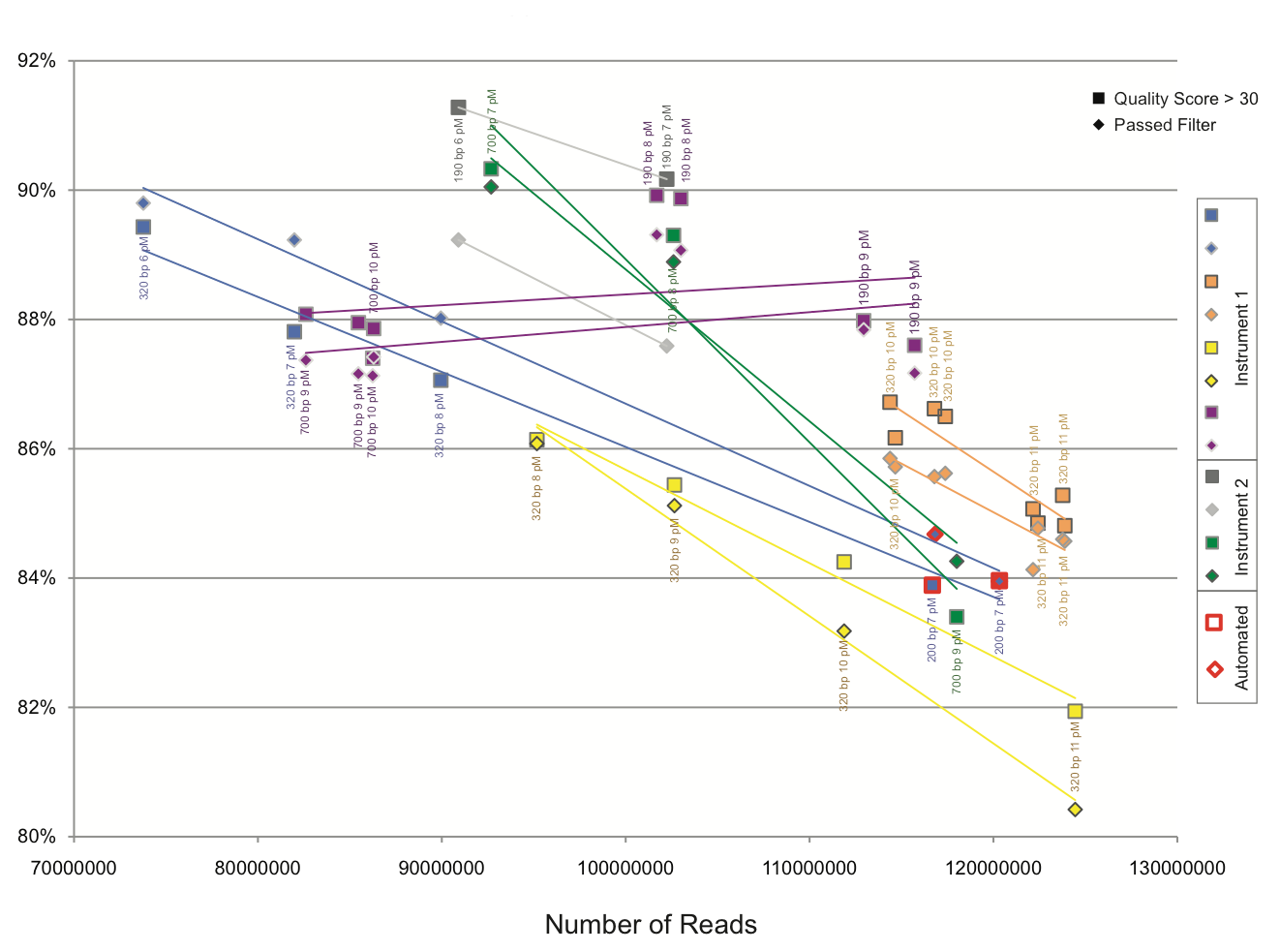

Supplement: Figure S1 — Effect of different clustering parameters and instrument runs. Passed filter rates and percentage of PF read base calls that have quality scores above 30 for HiSeq 2000 lanes with manually and automatically (red edge) prepared spruce samples. The colors of the markers denote different instrument runs. Insert size and concentration used for the cluster generation can be found in the label for each pair of data points. (TIF) [file pone.0019119.s001.tif]

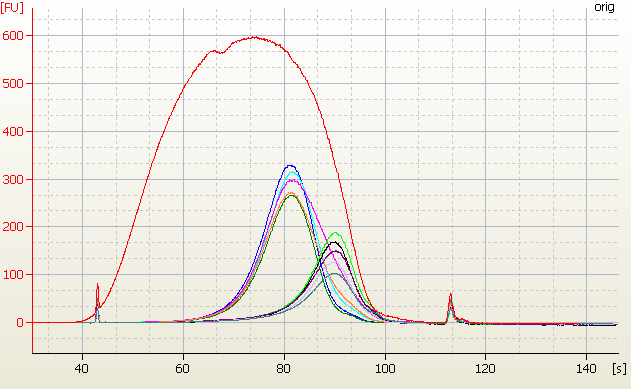

Supplement: Figure S2 — Robustness of the automatic size selection method. Two intervals (500 bp and 600 bp) were size selected and repeated five times. (TIF) [file pone.0019119.s002.tif]
