# Supplementary material for: Large Scale Library Generation for High Throughput Sequencing
Source: PLoS One. 2011 Apr 27;6(4):e19119. doi: 10.1371/journal.pone.0019119 (PMC3083417; doi:10.1371/journal.pone.0019119)
Supplement: Table S1 — PEG concentration of the two solutions used for each size interval in Figure 1. (DOCX) [file pone.0019119.s003.docx]

Table S1:

| Aimed average size: | PEG conc. (%) in first precipitation: | PEG conc. (%) in second precipitation: |
| --- | --- | --- |
| 200 | 11,6 | 12,6 |
| 300 | 9,8 | 10,6 |
| 400 | 8,7 | 9,5 |
| 500 | 7,9 | 8,6 |
| 600 | 7,4 | 8,0 |
| 700 | 6,9 | 7,5 |
