# Supplementary material for: Large Scale Library Generation for High Throughput Sequencing
Source: PLoS One. 2011 Apr 27;6(4):e19119. doi: 10.1371/journal.pone.0019119 (PMC3083417; doi:10.1371/journal.pone.0019119)
Supplement: Table S2 — Parameters and results for the 25 extra lanes included in Figure 2 and Figure S2. (DOCX) [file pone.0019119.s004.docx]

Table S2:

| Lane | Sample | Note | I.-size | Conc. (pM) | Cluster Density | reads | PF | >Q30 |
| --- | --- | --- | --- | --- | --- | --- | --- | --- |
| 1 | Spruce | Manual | 320 | 8 | 516 | 95171516 | 86,1% | 86,1% |
| 2 | Spruce | Manual | 320 | 9 | 557 | 102651853 | 85,1% | 85,4% |
| 3 | Spruce | Manual | 320 | 10 | 607 | 111880037 | 83,2% | 84,3% |
| 4 | Spruce | Manual | 320 | 11 | 675 | 124446189 | 80,4% | 81,9% |
| 5 | Spruce | Manual | 320 | 10 | 637 | 117381367 | 85,6% | 86,5% |
| 6 | Spruce | Manual | 320 | 10 | 634 | 116792045 | 85,6% | 86,6% |
| 7 | Spruce | Manual | 320 | 10 | 621 | 114376572 | 85,9% | 86,7% |
| 8 | Spruce | Manual | 320 | 10 | 622 | 114662098 | 85,7% | 86,2% |
| 9 | Spruce | Manual | 320 | 11 | 672 | 123880632 | 84,6% | 84,8% |
| 10 | Spruce | Manual | 320 | 11 | 663 | 122152559 | 84,1% | 85,1% |
| 11 | Spruce | Manual | 320 | 11 | 664 | 122417600 | 84,8% | 84,9% |
| 12 | Spruce | Manual | 320 | 11 | 672 | 123765075 | 84,6% | 85,3% |
| 13 | Spruce | Manual | 700 | 7 | 503 | 92683150 | 90,1% | 90,3% |
| 14 | Spruce | Manual | 700 | 8 | 557 | 102601762 | 88,9% | 89,3% |
| 15 | Spruce | Manual | 700 | 9 | 640 | 118004112 | 84,3% | 83,4% |
| 16 | Spruce | Manual | 190 | 6 | 493 | 90904386 | 89,2% | 91,3% |
| 17 | Spruce | Manual | 190 | 7 | 555 | 102233344 | 87,6% | 90,2% |
| 18 | Spruce | Manual | 700 | 9 | 464 | 85462350 | 87,2% | 88,0% |
| 19 | Spruce | Manual | 700 | 9 | 448 | 82608061 | 87,4% | 88,1% |
| 20 | Spruce | Manual | 700 | 10 | 468 | 86294052 | 87,4% | 87,9% |
| 21 | Spruce | Manual | 700 | 10 | 468 | 86242231 | 87,1% | 87,4% |
| 22 | Spruce | Manual | 190 | 8 | 552 | 101688258 | 89,3% | 89,9% |
| 23 | Spruce | Manual | 190 | 8 | 559 | 103001796 | 89,1% | 89,9% |
| 24 | Spruce | Manual | 190 | 9 | 613 | 112943121 | 87,8% | 88,0% |
| 25 | Spruce | Manual | 190 | 9 | 628 | 115714408 | 87,2% | 87,6% |
